# The effects of neutral argon plasma versus electrocoagulation on tissue in advanced-stage ovarian cancer: a case series

**DOI:** 10.1186/s13048-022-01070-5

**Published:** 2022-12-29

**Authors:** Gatske M. Nieuwenhuyzen-de Boer, Nick J. van de Berg, Xu Shan Gao, Patricia C. Ewing-Graham, Heleen J. van Beekhuizen

**Affiliations:** 1 Department of Gynecologic Oncology, Erasmus MC Cancer Institute, P.O. Box 2040, 3000CA Rotterdam, The Netherlands; 2 Department of Obstetrics and Gynecology, Albert Schweitzer Hospital, Dordrecht, The Netherlands; 3 Department of Biomechanical Engineering, Delft University of Technology, Delft, The Netherlands; 4 Department of Pathology, Erasmus University MC, University Medical Center, Rotterdam, The Netherlands

**Keywords:** Ovarian cancer, Ovarian neoplasms, Cytoreductive surgery, Histology, Thermal damage depth, Neutral argon plasma, PlasmaJet®, Electrocoagulation

## Abstract

**Background:**

The aim of surgery for advanced-stage ovarian cancer is a complete cytoreduction, because this is the most important independent prognostic factor for prolonged survival. Yet this can be difficult to achieve when there are micrometastases on the intestinal mesentery or intestines. The PlasmaJet device is an instrument to remove these micrometastases, but little is known about the depth of damage in human tissue compared to electrocoagulation devices.

**Methods:**

A prospective study was performed for the ex-vivo comparison of the histological depth of thermal damage of neutral argon plasma (PlasmaJet®) and electrocoagulation devices, in a series of 106 histological slides of 17 advanced-stage ovarian cancer patients. Depending on the tissue types resected during complete cytoreductive surgery, samples were collected from reproductive organs (uterus, ovaries), intestines (ileum, colon, rectum) and omentum, intestinal mesentery and peritoneum.

**Results:**

Average thermal damage depth was 0.15 mm (range 0.03–0.60 mm) after use of neutral argon plasma and 0.33 mm (range 0.08–1.80 mm) after use of electrocoagulation (*p* < 0.001). Greater disruption of the tissue surface was often observed after electrocoagulation.

**Conclusion:**

Our case series suggests that the use of neutral argon plasma during cytoreductive surgery produces significantly less thermal damage than electrocoagulation treatment. It is therefore considered a thermally safe alternative, aiding in the achievement of cytoreductive surgery.

## Synopsis

In this case series in ovarian cancer patients, the histological depth of thermal damage is compared after the use of PlasmaJet and electrocoagulation devices. PlasmaJet treatment produces significantly less thermal damage than electrocoagulation treatment.

## Introduction

Ovarian cancer is the fifth leading cause of cancer death in women, with over 14.000 cases yearly in the United States [1]. Surgery to remove all visible tumor in combination with chemotherapy is the most common therapy for advanced-stage ovarian cancer (ASOC). The aim of surgery for ASOC is complete cytoreductive surgery (CRS) to no visible disease, as it leads to longer progression free survival (PFS) and overall survival (OS) [2, 3]. However, it can be challenging to achieve complete CRS in patients with micrometastases on the intestinal mesentery, intestines or if the tumor reaches great vessels. These patients often need radical surgery, including upper abdominal surgery and bowel resection which increases the risk of complications [4, 5].

The use of Neutral Argon Plasma (PlasmaJet®, Plasma Surgical, Roswell, GA, USA ) is a relatively new device for CRS in ASOC management which may contribute to tissue ablation near to vulnerably locations to improve the percentage complete CRS [6]. The PlasmaJet emits a high-energy jet of argon plasma for direct tissue effects and is able to cut or vaporize small tumor foci [7]. During this process light, heat and kinetic energy are emitted.

When introducing new instruments, potential hazards of thermal damage like spontaneous bowel perforations and strictures, must be included in the risk analysis. Studies and reviews have indicated that the device is safe and effective for use in the surgical treatment of benign and malignant gynecological conditions with regard to postoperative complications [8–11]. The current insight in histological outcome of thermal tissue effects is based on case reports and two studies involving in-vivo porcine models [6, 7]. Studies quantified thermal tissue effects, and one described occurrence of postsurgical adhesions [7]. Only two studies directly compared the PlasmaJet to a second thermal coagulator and presented comparative data on thermal effects [8, 9].

The aim of our study was to assess the depth of the thermal tissue effects of the PlasmaJet with those of the ERBE electrocoagulation device. A series of 106 histological slides are described in which the depth of thermal damage was measured in various healthy tissues from reproductive organs (uterus, ovaries), intestines (ileum, colon, rectum) and omentum, intestinal mesentery and peritoneum.

## Materials and methods

The patients whose tissue is examined in this case series were included in the PlaComOv-study. The PlaComOv-study is a multicenter randomized controlled trial and compares the rates of complete CRS of patients with ASOC operated with the standard use of electrocoagulation (control group) with patients operated with the adjuvant use of PlasmaJet (intervention group) [12].

The study is carried out in accordance with the standards outlined in the Declaration of Helsinki. Ethics committee approval was granted. All patients were given both verbal and written information by their gynecologist before surgery. Informed consent to allow use of the data for analysis was obtained.

The patients selected for this case series underwent interval CRS because of ASOC between 2018 and 2020. All patients diagnosed with a high grade serous epithelial adenocarcinoma received neoadjuvant chemotherapy consisted of three cycles of intravenously carboplatin (area under the curve of 6 mg per milliliter per minute) and paclitaxel (175 mg per square meter of body-surface area) with a duration of three weeks for each cycle [13]. All cases were randomized to the intervention group.

### Surgical procedures

Patients randomized to the intervention group (adjuvant use of PlasmaJet during surgery) could be included in this study. Surgery was performed in the Erasmus MC so that the use of the PlasmaJet and ERBE coagulation proceeded in the same way. According to standard cytoreductive surgery for ovarian cancer, hysterectomy, adnexectomy and omentectomy was performed and if required a bowel resection was done in order to remove all visible tumour. Histological examination of the bowel could only be performed if there was visible tumor requiring bowel resection. Because of the research question to compare the depth of tissue infiltration, we only used tissue in which no visible vital tumor was seen. The tissue of interest was processed with the PlasmaJet and with the ERBE electrocoagulation device before removal and was marked with sutures to enable identification for histological research.

The PlasmaJet device was used at power setting 10, for durations ranging between 3 and 4 s. The distance to tissue ranged between 5 and 10 mm. The thermal effects were compared to those of an ERBE electrosurgical unit (VIO 300 D/S, Tübingen, DE), used at a power of 45 W, effect 4–5 for 1–2 s.

### Histological analysis

All gross specimens for histological analysis were handled according to the local protocol and transported to the laboratory within one hour after surgery. Blocks were taken from the areas marked with sutures during the operation as having been treated with PlasmaJet or electrocoagulation. The depth of thermal damage was measured on haematoxylin and eosin stained slides from the formalin-fixed, paraffin-embedded tissue blocks. To ensure uniform assessment, measurements were performed by a single blinded experienced gynecopathologist. Thermal damage was quantified as the largest orthogonal distance from the surface to the first layer of unchanged tissue. All slides were reviewed and the measurements checked by two of the authors (GN, PE). For each observed zone three different histological regions were studied in order to average our measurement.

### Statistical analysis

For statistical comparison, tissue types were grouped into reproductive organs (uterus, ovaries), intestines (ileum, colon, rectum) and omentum, intestinal mesentery and peritoneum. The results obtained with the PlasmaJet and ERBE electrocoagulation devices were compared in boxplots and evaluated with a Kruskal Wallis, and with Mann–Whitney U tests, using a significance level (ɑ) of 0.05.

## Results

A total of 106 histological regions were studied to assess destruction and thermal damage in tissue samples of 17 women who underwent interval CRS (Table 1). In the intestinal mesentery the measurement was not possible after electrocoagulation, as tissues were destroyed totally by using electrocoagulation for one second.

**Table 1:** Mean (range) depth of thermal damage after use of PlasmaJet and electrocoagulation devices, sorted by tissue type. *N* = number of samples

Tissue type		PlasmaJet, damage (mm)	*n*	Electrocoagulation, damage (mm)	*n*
**Reproduction organs**	Uterus	0.08 (0.03–0.20)	8	0.36 (0.15–0.90)	6
	Ovaries	0.43	1	1.09 (0.38–1.80)	2
**Intestines**	Ileum	0.11 (0.05–0.20)	6	0.44 (0.20–0.70)	5
	Colon	0.13 (0.07–0.20)	8	0.19 (0.10–0.30)	7
	Rectum	0.17 (0.15–0.18)	2	0.09 (0.08–0.10)	2
	Omentum	0.15 (0.05–0.30)	16	0.23 (0.08–0.55)	12
	Intestinal mesentery	0.50	1	-	-
	Peritoneum	0.17 (0.03–0.60)	15	0.35 (0.10-1.00)	13

**Fig. 1:**
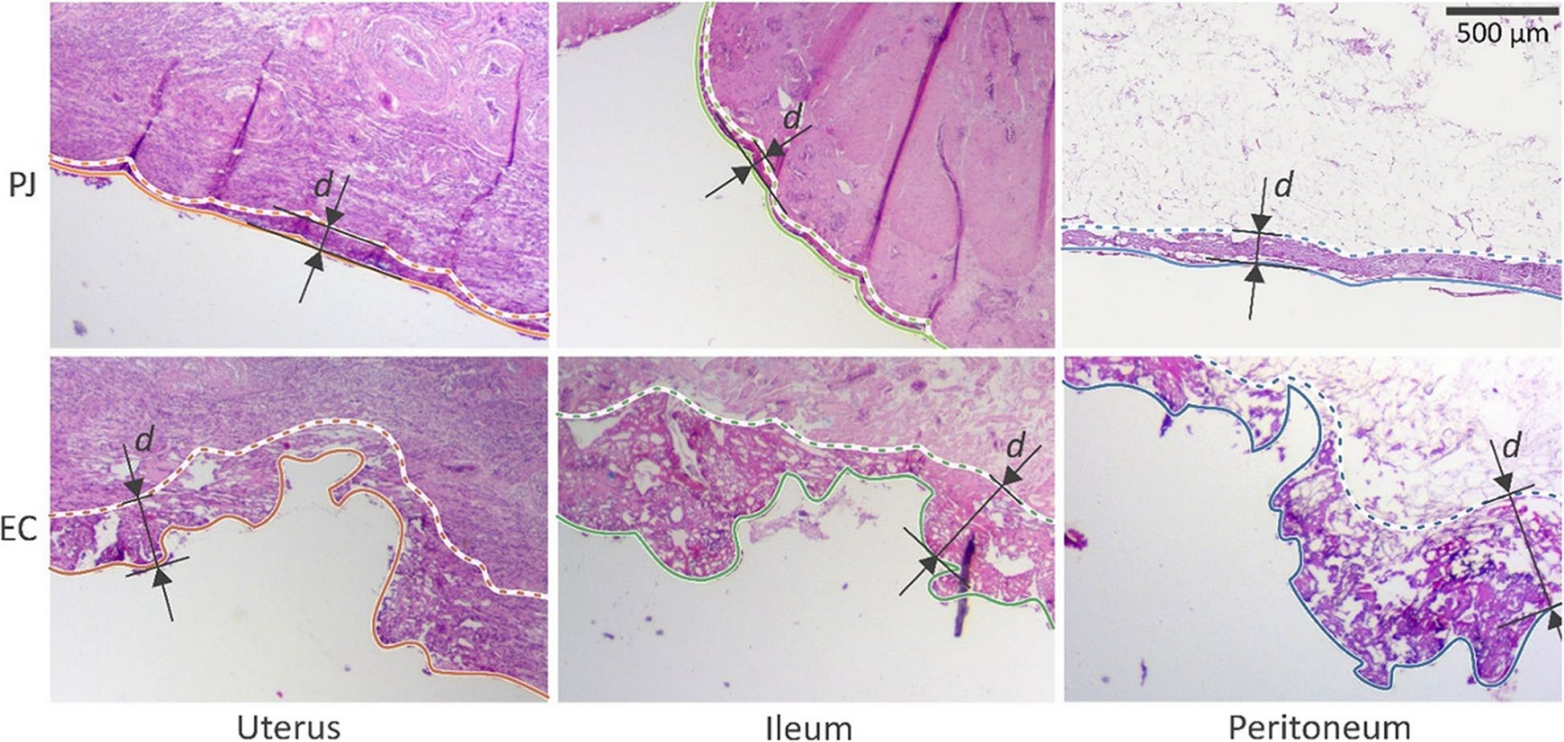
Exemplar histological images of thermal damage after use of the PlasmaJet (PJ) or ERBE electrocoagulation (EC). Depth of thermal damage (d) is indicated by arrows

Representative histological images for the three main groups, i.e. reproductive organs, intestines and ‘others’ (omentum, intestinal mesentery and peritoneum) are shown in Fig. 1. The measurements were taken from the surface of the tissue to the depth where the tissue was not damaged. In the evaluation of the tissue treated with electrocoagulation, it was noted that this tissue has an irregular surface (Fig. 1). The damage caused by the disappearance of this tissue has not been included in the measurements because this is also visible macroscopically.

**Fig. 2:**
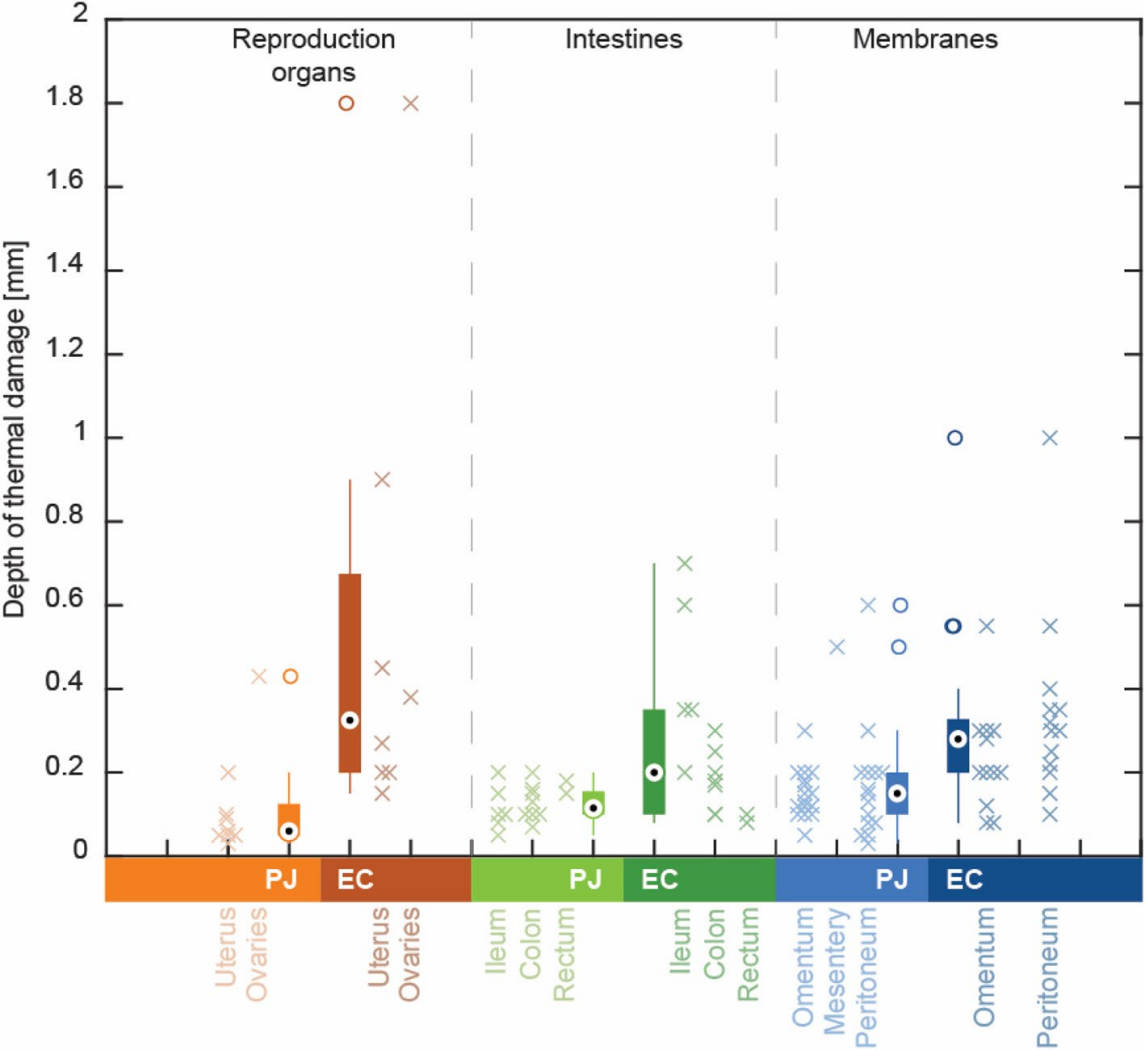
Comparison of depth of thermal damage after use of PlasmaJet (PJ) or ERBE electrocoagulation (EC) devices, for various tissue types, grouped in reproductive organs, intestines and membranes

The data are summarized in boxplots in Fig. 2. The mean of the depth of thermal damage after use of PlasmaJet was 0.15 mm (range 0.03–0.60 mm). After use of an electrocoagulation device the mean depth of thermal damage was 0.33 mm (range 0.08–1.80 mm). After clustering, the mean ± standard deviation of depth of thermal damage was 0.12 ± 0.13 mm (PJ) and 0.54 ± 0.56 mm (EC) in reproductive organs, 0.13 ± 0.05 mm (PJ) and 0.26 ± 0.19 mm (EC) in intestines, and 0.17 ± 0.12 mm (PJ) and 0.29 ± 0.19 mm (EC) in the group of omentum, intestinal mesentery and peritoneum. These groups were compared using the Kruskal Wallis test (.*p* < 0.001), and the Mann-Whitney U test. These showed that thermal damage was consistently lower after PlasmaJet than after electrocoagulation, i.e. for reproductive organs (*p* = 0.003), intestines (*p* = 0.013) and in the group of omentum, intestinal mesentery and peritoneum (*p* < 0.001).

### Discussion

In our case series of 106 histological slides of 17 advanced-stage ovarian cancer patients, mean thermal damage depth was significantly lower when tissue was treated with Neutral Argon Plasma, 0.15 mm (range 0.03–0.60 mm) than with electrocoagulation, 0.33 mm (range 0.08–1.80 mm, *p* < 0.001). Tissue treated with the PlasmaJet often showed a thin regular affected layer along the surface. In contrast, tissue treated with electrocoagulation, the tissue was rugged and disrupted (Fig 1).

Our data correlates well with the findings of Roman et al. [14] (mean: 0.145 mm), Sonoda et al. [11] (mean: 0.13 mm), and the shorter exposure times tested by Madhuri et al. [15] (mean 0.8 mm). Deb et al. report damage values in the 0.5–0.6 mm range [8]. However, it is unclear whether they used comparable histological definitions for the depth of thermal effects.

Neutral Argon Plasma enables ablation with a highly controlled tissue effect, allowing treatment of sites previously considered untreatable, e.g. metastases on the intestinal mesentery and serosa of intestines [16]. With short application times, energy dissipation to deeper structures can be avoided [8]. The PlasmaJet device also eliminates some of the risks of electrosurgical devices because no electrical current passes through the tissue. As the PlasmaJet tip is continuously cooled by circulating water, the risk of inadvertent burns is minimized.

The histological slides in these case series confirm minimal tissue damage from the PlasmaJet on the gut. In particular for the gut, little data on the thermal infiltration depth are publicly available. Until now, it was not possible to remove tumor on the intestines and leave the intestines in situ due to the depth of infiltration of electrocoagulation devices. These measurements show that the infiltration damage from using the PlasmaJet is less than after using electrocoagulation.

A possible explanation for the variations infiltration damage could be related to the difference between the devices. The PlasmaJet emits a high-energy beam of argon plasma for direct tissue effects. Electrocoagulation requires the tissue to be heated rapidly by a required current density achieved by short electrical arcs (sparks) that occur at peak voltages from around 200 V between the electrode and the tissue. The absence of sparks and uncontrolled bursts of energy entering the tissue might explain the reduced deep tissue injury when using the PlasmaJet.

In clinical practice, the effect of less tissue infiltration by using the PlasmaJet may also be reflected in the quality of life. Patients who had surgery using the PlasmaJet showed a higher quality of life six months after the procedure [17]. A possible explanation could be that less tissue damage results in a different process of tissue repair and regeneration (inflammation, proliferation by fibrogenesis and angiogenesis and remodeling). In contrast, when there is more tissue damage, it will proceed differently. The surgeon must be aware of the effect of the instrument used, especially when extensive peritoneal stripping is involved in the surgery.

However, in other tissue types (e.g. intestinal mesentery) thermal effects may remain unclear, as sample sizes relied on the clinical necessity of tissue removal. As a result, clustering of data was required. It should also be noted that knowledge about tissue damage is particularly relevant for those tissues that remain in the patient. In this study, the tissues of uterus, adnexa and omentum were included for comparison with data in literature.

A difficulty of our study was the assessment of the depth of histological tissue damage after electrocoagulation treatment, due to disrupted and irregular tissue surfaces. In our analysis, we measured damage as the thickness of the layer of altered tissue (Fig. 1). An alternative approach could be to include the depth of disruption, vaporization and charring formed by electrocoagulation, when measuring the depth of effect. This approach would significantly increase the difference seen after using PlasmaJet and electrocoagulation devices. The importance of including exemplary histological images to illustrate the approach used should be emphasized.

The PlasmaJet device was used at power setting 10, for durations ranging between 3 and 4 s. For the PlasmaJet device, depth of vaporization was found to increase with exposure time [11, 15]. Possibly, vaporization depth is also weakly related to the power setting [11]. Lateral thermal spread is likely to be dependent on exposure time, but not on power setting [9, 11, 15]. However, in an in-vitro setting, Deb et al. [8] found no effect of exposure time or power setting on lateral spread or width of the affected zone.

In general, our results correspond to other studies and have demonstrated that the depth of thermal damage of the use of Neutral Argon Plasma remains superficial. Situations where the use of PlasmaJet could clearly be beneficial include the treatment of tissue that would otherwise remain untreated and the treatment of micrometastasis thereby avoiding bowel resection.

## Conclusion

Based on our case studies we conclude that a Neutral Argon Plasma device produces significantly less thermal damage than an electrocoagulation device. The PlasmaJet is therefore a thermally safe device that can be used to aid during cytoreductive surgery in patients with advanced-stage ovarian cancer. We recommend subsequent research to evaluate whether microscopic deposits of vital tumor tissue remain on relevant organs such as bowel after vaporization.

## Abbreviations

ASOC: Advanged-stage Ovarian Cancer
CAP: Cold Atmospheric Plasma
CRS: Cytoreductive Surgery
EC: Electrocoagulation
OS: Overall Survival
PFS: Progression-Free Survival
PJ: PlasmaJet
